# Supplementary material for: Vaccine effectiveness against SARS-CoV-2 infection or COVID-19 hospitalization with the Alpha, Delta, or Omicron SARS-CoV-2 variant: A nationwide Danish cohort study
Source: PLoS Med. 2022 Sep 1;19(9):e1003992. doi: 10.1371/journal.pmed.1003992 (PMC9436060; doi:10.1371/journal.pmed.1003992)
Supplement: S2 Table — (DOCX) [file pmed.1003992.s002.docx]

Table S2. Overview of results from previous studies

|  | **Age of study population** | **Vaccine type** | **Number of**  **vaccine doses** | **Outcomes** | **Estimate** | **SARS-CoV-2 variant** | **Vaccine protection (95% CI)** | **Vaccine protection by time since vaccination** |
| --- | --- | --- | --- | --- | --- | --- | --- | --- |
| Barda N et al. [1] | 12 years or above | BNT162b2 mRNA | Three doses compared to two doses | Admission to hospital | VE | Delta | 93% (88; 97) | Not available |
|  |  |  |  | Severe disease |  |  | 92% (82; 97) |  |
| Lopez Bernal et al. [2] | 16 years or above | BNT162b2 mRNA | Two doses compared to unvaccinated | Symptomatic disease | VE | Alpha | 93.7% (91.6; 95.3) | Not available |
|  |  |  |  |  |  | Delta | 88.0% (85.3; 90.1) |  |
| Pouwels et al. [3] | 18 years or above | BNT162b2 mRNA | Two doses compared to unvaccinated | All infections | VE | Alpha | 78% (68; 84) | Estimates available for 14, 30, 60 and 90 days since the second dose |
|  |  |  |  |  |  | Delta | 80% (77; 83) |  |
|  |  |  |  | Self-reported symptoms |  | Alpha | 97% (96; 98) |  |
|  |  |  |  |  |  | Delta | 84% (82; 86%) |  |
| Accorsi et al. [4] | 18 years or above | BNT162b2 mRNA or mRNA-1273 | Three doses compared to unvaccinated | Symptomatic SARS-CoV-2 Infection | OR | Delta | 0.065 (0.059; 0.071) | Estimates available for 0-11 months since second dose |
|  |  |  |  |  |  | Omicron | 0.33 (0.31; 0.35) |  |
|  |  |  | Three doses compared to two doses |  |  | Delta | 0.16 (0.14; 0.17) |  |
|  |  |  |  |  |  | Omicron | 0.34 (0.32; 0.36) |  |
| Andrews et al. [5] | 18 years or above | BNT162b2 mRNA | Two doses compared to unvaccinated | Symptomatic Disease | VE | Delta | 90.9% (89.6; 92.0) | Estimates available for 2 to ≥25 weeks since the second dose and 1 to ≥10 weeks since the third dose |
|  |  |  |  |  |  | Omicron | 65.5% (63.9; 67.0) |  |
|  |  |  | Three doses compared to unvaccinated |  |  | Delta | 92.3% (91.6; 92.9) |  |
|  |  |  |  |  |  | Omicron | 66.9% (66.1; 67.6) |  |
|  |  | mRNA-1273 | Two doses compared to unvaccinated |  |  | Delta | 94.5% (90.5; 96.9) |  |
|  |  |  |  |  |  | Omicron | 75.1% (70.8; 78.7) |  |
|  |  |  | Three doses compared to unvaccinated |  |  | Delta | 95.3% (92.1; 97.2) |  |
|  |  |  |  |  |  | Omicron | 68.1% (65.6; 70.5) |  |
| Tseng et al. [6] | 18 years or above | mRNA-1273 | Two doses compared to unvaccinated | Infection | VE | Delta | 80.2% (68.2; 87.7) | Estimates available for 14 to >270 days since the second dose and 14 to >60 days since the third dose |
|  |  |  |  |  |  | Omicron | 44.0% (35.1; 51.6) |  |
|  |  |  |  | Hospitalization |  | Delta | 99.0% (93.3; 99.9) |  |
|  |  |  |  |  |  | Omicron | 84.5% (23.0; 96.9) |  |
|  |  |  | Three doses compared to unvaccinated | Infection |  | Delta | 93.7% (92.2; 94.9) |  |
|  |  |  |  |  |  | Omicron | 71.6% (69.7; 73.4) |  |
|  |  |  |  | Hospitalization |  | Delta | 99.7% (96.5; 100.0) |  |
|  |  |  |  |  |  | Omicron | 99.2% (76.3; 100.0) |  |
| Lauring et al. [7] | 18 years or above | BNT162b2 mRNA or mRNA-1273 | Two doses compared to unvaccinated | Covid-19 hospital admission | VE | Alpha | 85% (82; 88) | For the Alpha and Delta variants, estimates $\leq$150 days and >150 days since the second dose are available |
|  |  |  |  |  |  | Delta | 85% (83; 87) |  |
|  |  |  |  |  |  | Omicron | 65% (51; 75) |  |
|  |  |  | Three doses compared to unvaccinated |  |  | Delta | 94% (92; 95) |  |
|  |  |  |  |  |  | Omicron | 86% (77; 91) |  |
| Nyberg et al. [8] | All ages | BNT162b2 mRNA or mRNA-1273 | Two doses | Hospital  admission up  to 14 days after  positive test | HR | Delta | 0.45 (0.29-0.70) | Less than 2 to ≥20 weeks since the second dose and less than 2 to ≥12 weeks since the third dose |
|  |  |  |  |  |  | Omicron | 0.40 (0.31-0.51) |  |
|  |  |  | Three doses |  |  | Delta | 0.13 (0.11-0.15) |  |
|  |  |  |  |  |  | Omicron | 0.26 (0.23-0.29) |  |

1. Barda N, Dagan N, Cohen C, Hernán MA, Lipsitch M, Kohane IS, et al. Effectiveness of a third dose of the BNT162b2 mRNA COVID-19 vaccine for preventing severe outcomes in Israel: an observational study. Lancet. 2021;398(10316):2093-100. Epub 2021/10/29. doi: 10.1016/S0140-6736(21)02249-2. PubMed PMID: 34756184.

2. Lopez Bernal J, Andrews N, Gower C, Gallagher E, Simmons R, Thelwall S, et al. Effectiveness of Covid-19 Vaccines against the B.1.617.2 (Delta) Variant. N Engl J Med. 2021;385(7):585-94. Epub 2021/07/22. doi: 10.1056/NEJMoa2108891. PubMed PMID: 34289274; PubMed Central PMCID: PMCPMC8314739.

3. Pouwels KB, Pritchard E, Matthews PC, Stoesser N, Eyre DW, Vihta KD, et al. Effect of Delta variant on viral burden and vaccine effectiveness against new SARS-CoV-2 infections in the UK. Nat Med. 2021;27(12):2127-35. Epub 2021/10/16. doi: 10.1038/s41591-021-01548-7. PubMed PMID: 34650248; PubMed Central PMCID: PMCPMC8674129.

4. Accorsi EK, Britton A, Fleming-Dutra KE, Smith ZR, Shang N, Derado G, et al. Association Between 3 Doses of mRNA COVID-19 Vaccine and Symptomatic Infection Caused by the SARS-CoV-2 Omicron and Delta Variants. JAMA. 2022;327(7):639-51. doi: 10.1001/jama.2022.0470.

5. Andrews N, Stowe J, Kirsebom F, Toffa S, Rickeard T, Gallagher E, et al. Covid-19 Vaccine Effectiveness against the Omicron (B.1.1.529) Variant. N Engl J Med. 2022. Epub 2022/03/07. doi: 10.1056/NEJMoa2119451. PubMed PMID: 35249272; PubMed Central PMCID: PMCPMC8908811.

6. Tseng HF, Ackerson BK, Luo Y, Sy LS, Talarico CA, Tian Y, et al. Effectiveness of mRNA-1273 against SARS-CoV-2 Omicron and Delta variants. Nat Med. 2022. Epub 2022/02/22. doi: 10.1038/s41591-022-01753-y. PubMed PMID: 35189624.

7. Lauring AS, Tenforde MW, Chappell JD, Gaglani M, Ginde AA, McNeal T, et al. Clinical severity of, and effectiveness of mRNA vaccines against, covid-19 from omicron, delta, and alpha SARS-CoV-2 variants in the United States: prospective observational study. BMJ. 2022;376:e069761. doi: 10.1136/bmj-2021-069761.

8. Nyberg T, Ferguson NM, Nash SG, Webster HH, Flaxman S, Andrews N, et al. Comparative analysis of the risks of hospitalisation and death associated with SARS-CoV-2 omicron (B.1.1.529) and delta (B.1.617.2) variants in England: a cohort study. Lancet. 2022;399(10332):1303-12. Epub 2022/03/20. doi: 10.1016/s0140-6736(22)00462-7. PubMed PMID: 35305296; PubMed Central PMCID: PMCPMC8926413.
