# Supplementary material for: Vaccine effectiveness against SARS-CoV-2 infection or COVID-19 hospitalization with the Alpha, Delta, or Omicron SARS-CoV-2 variant: A nationwide Danish cohort study
Source: PLoS Med. 2022 Sep 1;19(9):e1003992. doi: 10.1371/journal.pmed.1003992 (PMC9436060; doi:10.1371/journal.pmed.1003992)
Supplement: S3 Table — (DOCX) [file pmed.1003992.s003.docx]

Table S3. Unadjusted vaccine effectiveness of two doses BNT162b2 mRNA or mRNA-1273 against SARS-CoV-2 infection with the Alpha, Delta or Omicron variant by age groups (12-59 years and 60 years or above)

|  | **Alpha** | | | | | **Delta** | | | | | **Omicron** | | | | |
| --- | --- | --- | --- | --- | --- | --- | --- | --- | --- | --- | --- | --- | --- | --- | --- |
|  | **Population** | **Person-years** | **Cases** | **VE** | **95% CI** | **Population** | **Person-years** | **Cases** | **VE** | **95% CI** | **Population** | **Person-years** | **Cases** | **VE** | **95% CI** |
| **12-59 years** |  |  |  |  |  |  |  |  |  |  |  |  |  |  |  |
| Unvaccinated |  |  |  |  |  | 961,947 | 143,400 | 43,581 | 1 (reference) |  | 179,417 | 15,470 | 96,160 | 1 (reference) |  |
| Time since vaccination |  |  |  |  |  |  |  |  |  |  |  |  |  |  |  |
| 14-30 days |  |  |  |  |  | 1,600,382 | 74,050 | 1,624 | 92.3 | 91.9; 92.7 | 61,480 | 2,147 | 8,252 | 36.1 | 34.6; 37.5 |
| 31-60 days |  |  |  |  |  | 1,598,449 | 129,640 | 3,718 | 88.8 | 88.4; 89.2 | 63,919 | 3,019 | 16,502 | 27.0 | 25.7; 28.2 |
| 61-90 days |  |  |  |  |  | 1,581,085 | 123,574 | 7,873 | 82.0 | 81.5; 82.4 | 57,597 | 2,158 | 9,759 | 26.8 | 25.3; 28.3 |
| 91-120 days |  |  |  |  |  | 1,400,902 | 85,887 | 14,950 | 71.8 | 71.2; 72.4 | 221,164 | 7,017 | 22,911 | 27.3 | 26.2; 28.4 |
| >120 days |  |  |  |  |  | 750,393 | 59,185 | 10,360 | 67.9 | 67.1; 68.6 | 1,076,044 | 56,890 | 276,075 | 9.5 | 8.9; 10.2 |
|  |  |  |  |  |  |  |  |  |  |  |  |  |  |  |  |
| **60 years or above** |  |  |  |  |  |  |  |  |  |  |  |  |  |  |  |
| Unvaccinated | 652,324 | 111,191 | 4,462 | 1 (reference) |  | 22,097 | 6,895 | 1,113 | 1 (reference) |  | 10,899 | 1,051 | 3,351 | 1 (reference) |  |
| Time since vaccination |  |  |  |  |  |  |  |  |  |  |  |  |  |  |  |
| 14-30 days | 407,513 | 16,797 | 78 | 90.8 | 88.3; 92.8 | 199,220 | 6,996 | 59 | 81.8 | 75.0; 86.7 | 1,341 | 48 | 96 | 37.9 | 23.9; 49.3 |
| 31-60 days | 323,594 | 16,190 | 144 | 80.1 | 76.1; 83.4 | 360,044 | 21,964 | 393 | 75.3 | 71.3; 78.8 | 1,290 | 61 | 136 | 37.1 | 25.3; 47.0 |
| 61-90 days | 116,308 | 7,274 | 119 | 65.7 | 58.2; 71.8 | 447,290 | 34,207 | 687 | 77.6 | 74.7; 80.1 | 1,072 | 43 | 103 | 25.0 | 8.8; 38.3 |
| 91-120 days | 58,348 | 3,467 | 49 | 77.3 | 69.6; 83.1 | 496,192 | 38,465 | 1,159 | 71.7 | 68.8; 74.2 | 1,750 | 77 | 173 | 23.5 | 10.9; 34.4 |
| >120 days | 35,699 | 1,445 | 23 | 68.6 | 50.0; 80.3 | 534,325 | 92,247 | 12,198 | 48.5 | 45.2; 51.6 | 45,835 | 1,781 | 4,722 | 4.2 | -0.3; 8.4 |

VE = vaccine effectiveness. CI = confidence intervals. VE estimates with underlying calendar time. Individuals were able to contribute follow-up time in more than one time category and (if vaccinated during the study period) to both the analysis of VE after two and three doses.
