# Supplementary material for: Vaccine effectiveness against SARS-CoV-2 infection or COVID-19 hospitalization with the Alpha, Delta, or Omicron SARS-CoV-2 variant: A nationwide Danish cohort study
Source: PLoS Med. 2022 Sep 1;19(9):e1003992. doi: 10.1371/journal.pmed.1003992 (PMC9436060; doi:10.1371/journal.pmed.1003992)
Supplement: S4 Table — (DOCX) [file pmed.1003992.s004.docx]

Table S4. Unadjusted vaccine effectiveness of three doses BNT162b2 mRNA or mRNA-1273 against SARS-CoV-2 infection with the Delta or Omicron variant by age groups (18-59 years and 60 years or above)

|  | **Delta** | | | | | **Omicron** | | | | |
| --- | --- | --- | --- | --- | --- | --- | --- | --- | --- | --- |
|  | **Population** | **Person-years** | **Cases** | **VE** | **95% CI** | **Population** | **Person-years** | **Cases** | **VE** | **95% CI** |
| **18-59 years** |  |  |  |  |  |  |  |  |  |  |
| Unvaccinated | 757,872 | 110,504 | 34,412 | 1 (reference) |  | 144,946 | 12,652 | 74,627 | 1 (reference) |  |
| Time since vaccination |  |  |  |  |  |  |  |  |  |  |
| 14-30 days | 62,373 | 2,040 | 149 | 91.3 | 89.8; 92.6 | 880,288 | 34,122 | 103,590 | 57.1 | 56.7; 57.5 |
| 31-60 days | 19,416 | 502 | 69 | 82.0 | 77.2; 85.8 | 631,525 | 20,080 | 87,334 | 56.4 | 55.9; 56.9 |
| 61-90 days | 2,736 | 47 | 6 | 87.7 | 72.6; 94.5 | 91,301 | 5,505 | 15,540 | 58.2 | 57.5; 59.0 |
| 91-120 days |  |  |  |  |  | 44,271 | 1,329 | 5,322 | 57.8 | 56.6; 59.0 |
| >120 days |  |  |  |  |  | 4,259 | 144 | 580 | 60 | 56.6; 63.1 |
|  |  |  |  |  |  |  |  |  |  |  |
| **60 years or above** |  |  |  |  |  |  |  |  |  |  |
| Unvaccinated | 22,097 | 6,895 | 1,113 | 1 (reference) |  | 10,899 | 1,051 | 3,351 | Reference |  |
| Time since vaccination |  |  |  |  |  |  |  |  |  |  |
| 14-30 days | 81,470 | 3,031 | 156 | 85.9 | 83.2; 88.2 | 335,215 | 12,702 | 13,477 | 57.4 | 55.6; 59.2 |
| 31-60 days | 45,216 | 2,376 | 162 | 84.2 | 81.2; 86.7 | 390,527 | 21,077 | 34,137 | 57.7 | 56.2; 59.2 |
| 61-90 days | 14,015 | 225 | 26 | 83.7 | 75.5; 89.1 | 176,457 | 6,999 | 10,366 | 61.7 | 60.1; 63.2 |
| 91-120 days |  |  |  |  |  | 75,122 | 3,723 | 5,088 | 60.3 | 58.6; 62.0 |
| >120 days |  |  |  |  |  | 31,522 | 1,101 | 2,130 | 59.4 | 56.8; 61.9 |

VE = vaccine effectiveness. CI = confidence intervals. VE estimates with underlying calendar time. Individuals were able to contribute follow-up time in more than one time category and (if vaccinated during the study period) to both the analysis of VE after two and three doses.
