# Supplementary material for: Vaccine effectiveness against SARS-CoV-2 infection or COVID-19 hospitalization with the Alpha, Delta, or Omicron SARS-CoV-2 variant: A nationwide Danish cohort study
Source: PLoS Med. 2022 Sep 1;19(9):e1003992. doi: 10.1371/journal.pmed.1003992 (PMC9436060; doi:10.1371/journal.pmed.1003992)
Supplement: S5 Table — (DOCX) [file pmed.1003992.s005.docx]

Table S5. Unadjusted vaccine effectiveness of two doses BNT162b2 mRNA or mRNA-1273 against COVID-19 hospitalization following infection with the Alpha, Delta or Omicron variant by age groups (12-59 years and 60 years or above)

|  | **Alpha** | | | | | **Delta** | | | | | **Omicron** | | | | |
| --- | --- | --- | --- | --- | --- | --- | --- | --- | --- | --- | --- | --- | --- | --- | --- |
|  | **Population** | **Person-years** | **Cases** | **VE** | **95% CI** | **Population** | **Person-years** | **Cases** | **VE** | **95% CI** | **Population** | **Person-years** | **Cases** | **VE** | **95% CI** |
| **12-59 years** |  |  |  |  |  |  |  |  |  |  |  |  |  |  |  |
| Unvaccinated |  |  |  |  |  | 961,947 | 143,400 | 724 | 1 (reference) |  | 179,417 | 15,470 | 263 | 1 (reference) |  |
| Time since vaccination |  |  |  |  |  | 961,947 | 143,400 | 724 | 1 (reference) |  | 179,417 | 15,470 | 263 | 1 (reference) |  |
| 14-30 days |  |  |  |  |  |  |  |  |  |  |  |  |  |  |  |
| 31-60 days |  |  |  |  |  | 1,600,382 | 74,050 | 3 | 99.3 | 97.8; 99.8 | 61,480 | 2,147 | 1 | 97.2 | 80.3; 99.6 |
| 61-90 days |  |  |  |  |  | 1,598,449 | 129,640 | 7 | 99.0 | 97.9; 99.5 | 63,919 | 3,019 | 8 | 84.6 | 68.8; 92.4 |
| 91-120 days |  |  |  |  |  | 1,581,085 | 123,574 | 7 | 99.0 | 98.0; 99.5 | 57,597 | 2,158 | 8 | 78.6 | 56.8; 89.4 |
| >120 days |  |  |  |  |  | 1,400,902 | 85,887 | 25 | 96.7 | 95.0; 97.8 | 221,164 | 7,017 | 11 | 90.8 | 83.2; 95.0 |
|  |  |  |  |  |  | 750,393 | 59,185 | 77 | 83.2 | 78.5; 86.8 | 1,076,044 | 56,890 | 168 | 82.2 | 78.3; 85.3 |
| **60 years or above** |  |  |  |  |  |  |  |  |  |  |  |  |  |  |  |
| Unvaccinated |  |  |  |  |  |  |  |  |  |  |  |  |  |  |  |
| Time since vaccination | 652,324 | 111,191 |  | 1 (reference) |  | 22,097 | 6,895 | 276 | 1 (reference) |  |  |  |  |  |  |
| 14-30 days |  |  |  |  |  |  |  |  |  |  |  |  |  |  |  |
| 31-60 days | 407,513 | 16,797 | 4 | 96.8 | 91.2; 98.9 | 199,220 | 6,996 | 0 | 100.0 | * |  |  |  |  |  |
| 61-90 days | 323,594 | 16,190 | 15 | 84.7 | 73.1; 91.3 | 360,044 | 21,964 | 8 | 98.3 | 96.5; 99.2 |  |  |  |  |  |
| 91-120 days | 116,308 | 7,274 | 19 | 61.2 | 35.5; 76.6 | 447,290 | 34,207 | 18 | 98.2 | 97.0; 98.9 |  |  |  |  |  |
| >120 days | 58,348 | 3,467 | 5 | 81.4 | 53.6; 92.5 | 496,192 | 38,465 | 32 | 97.4 | 96.2; 98.2 |  |  |  |  |  |

VE = vaccine effectiveness. CI = confidence intervals. VE estimates with underlying calendar time. Individuals were able to contribute follow-up time in more than one time category and (if vaccinated during the study period) to both the analysis of VE after two and three doses.

* It was not possible within the model to estimate a 95% confidence interval for the estimated vaccine effectiveness against COVID-19 hospitalization with the Delta variant 14-30 days after the second dose as no COVID-19-related hospitalization were observed.
