# Supplementary material for: Vaccine effectiveness against SARS-CoV-2 infection or COVID-19 hospitalization with the Alpha, Delta, or Omicron SARS-CoV-2 variant: A nationwide Danish cohort study
Source: PLoS Med. 2022 Sep 1;19(9):e1003992. doi: 10.1371/journal.pmed.1003992 (PMC9436060; doi:10.1371/journal.pmed.1003992)
Supplement: S6 Table — (DOCX) [file pmed.1003992.s006.docx]

Table S6. Unadjusted vaccine effectiveness of three doses BNT162b2 mRNA or mRNA-1273 against COVID-19 hospitalization following infection with the Delta or Omicron variant by age groups (18-59 years and ≥60 years)

|  | **Delta** | | | | | **Omicron** | | | | |
| --- | --- | --- | --- | --- | --- | --- | --- | --- | --- | --- |
|  | **Population** | **Person-years** | **Cases** | **VE** | **95% CI** | **Population** | **Person-years** | **Cases** | **VE** | **95% CI** |
| **18-59 years** |  |  |  |  |  |  |  |  |  |  |
| Unvaccinated | 757,872 | 110,504 | 717 | 1 (reference) |  | 144,946 | 12,652 | 255 | 1 (reference) |  |
| Time since vaccination |  |  |  |  |  |  |  |  |  |  |
| 14-30 days | 62,373 | 2,040 | 4 | 85.9 | 61.8; 94.8 | 880,288 | 34,122 | 39 | 93.7 | 91.2; 95.6 |
| 31-60 days | 19,416 | 502 | 9 | -35.9 | -165.2; 30.3 | 631,525 | 20,080 | 41 | 90.0 | 85.6; 93.0 |
| 61-90 days |  |  |  |  |  | 91,301 | 5,505 | 37 | 65.7 | 51.5; 75.8 |
| 91-120 days |  |  |  |  |  | 44,271 | 1,329 | 32 | -23.2 | -82.9; 17.0 |
| >120 days |  |  |  |  |  | 4,259 | 144 | 17 | -438.8 | -804.3; -221.1 |
|  |  |  |  |  |  |  |  |  |  |  |
| **60 years or above** |  |  |  |  |  |  |  |  |  |  |
| Unvaccinated | 22,097 | 6,895 | 276 | 1 (reference) |  | 10,899 | 1,051 | 236 | 1 (Reference) |  |
| Time since vaccination |  |  |  |  |  |  |  |  |  |  |
| 14-30 days | 81,470 | 3,031 | 10 | 95.7 | 91.7; 97.7 | 335,215 | 12,702 | 65 | 97.6 | 96.9; 98.2 |
| 31-60 days | 45,216 | 2,376 | 32 | 85.4 | 78.5; 90.1 | 390,527 | 21,077 | 163 | 96.4 | 95.6; 97.1 |
| 61-90 days | 14,015 | 225 | 5 | 83.5 | 58.4; 93.5 | 176,457 | 6,999 | 159 | 89.8 | 87.5; 91.7 |
| 91-120 days |  |  |  |  |  | 75,122 | 3,723 | 213 | 74.4 | 69.2; 78.8 |
| >120 days |  |  |  |  |  | 31,522 | 1,101 | 95 | 61.2 | 48.0; 71.0 |

VE = vaccine effectiveness. CI = confidence intervals. VE estimates with underlying calendar time. Individuals were able to contribute follow-up time in more than one time category and (if vaccinated during the study period) to both the analysis of VE after two and three doses.
