# Supplementary material for: Vaccine effectiveness against SARS-CoV-2 infection or COVID-19 hospitalization with the Alpha, Delta, or Omicron SARS-CoV-2 variant: A nationwide Danish cohort study
Source: PLoS Med. 2022 Sep 1;19(9):e1003992. doi: 10.1371/journal.pmed.1003992 (PMC9436060; doi:10.1371/journal.pmed.1003992)
Supplement: Attachment — Submitted filename: gram.pdf [file pmed.1003992.s007.pdf]

This paper collates various databases to estimate vaccine effectiveness in Denmark.

I was asked for a statistical report and I interpret that to include all aspects of the design and conduct of the study.

Points of detail

Page 6 Was the age only available in 5-year bands or did the authors decide to categorise it? Categorising an essentially continuous variable wastes information (Altman and Royston, 2006; Royston et al., 2006) and leads to models which are often implausible as they predict the effect remaining flat within categories and then jumping to a new value at the category boundary.

Page 9 I think it would be better to include more explanation in the captions to the figures so that the figure plus caption are self–explanatory and do not need cross–referencing to the main text. Readers of the article on–line will see thumbnails and if they click on them they are not seeing the text. In the printed version there is no guarantee that the text is near the figure either. For instance in the case of Figure 1 some explanation of the blue jagged line would be good. I know we can puzzle it out for ourselves. Most of the other figures have rather sparse captions.

Page 9 I think some readers aged 60 and over might object to be called elderly. ‘Older age groups were . . . ’ might be kinder.

Page 14 Would it be helpful to present the results from these other studies in tabular form to aid comparison? If that is done then relevant comparator results from this article could be repeated there so readers can see the totality of evidence in one place.

Table 1 Somewhere we need an explanation for the choice of 60 years as the cut–point. Was this how the Danish health system decided to prioritise in the first wave of vaccinations?

Table 1 The caption defines an asterisk (*) but I do not see it in the body of the table. Apologies if this is just my ageing eyes.

Point of more substance

As time went by the number of unvaccinated people became quite small. From the point of view of analysis this is unimportant as the confidence intervals take care of the imprecision but who were these people? If they are very different from the vaccinated people then using them as a control group becomes problematic. Is there any Danish data on what mixture they were of people with needle phobia, people with auto–immune conditions worried about the effect on their primary diagnosis, people who knew Bill Gates was going to implant a micro–chip in them, . . . ? Obviously this would have to come from outside the present study. In my country early on the true statement that we had no data on pregnant women was translated into the advice which many pregnant women received not to get vaccinated. This had tragic effects. Did this also happen in Denmark?

Summary

Mostly requests for more detail and clarification.

Michael Dewey

References

D G Altman and P Royston. The cost of dichotomising continuous variables. *British Medical Journal*, 332:1080, 2006.

P Royston, D G Altman, and W Sauerbrei. Dichotomizing continuous predictors in multiple regression: a bad idea. *Statistics in Medicine*, 25:127–141, 2006.
