# Supplementary material for: Vaccine effectiveness against SARS-CoV-2 infection or COVID-19 hospitalization with the Alpha, Delta, or Omicron SARS-CoV-2 variant: A nationwide Danish cohort study
Source: PLoS Med. 2022 Sep 1;19(9):e1003992. doi: 10.1371/journal.pmed.1003992 (PMC9436060; doi:10.1371/journal.pmed.1003992)
Supplement: Attachment — Submitted filename: Review_Vaccine effectiveness against SARS-CoV-2 infection and COVID-19-related hospitalization with the Alpha, Delta, and Omicron SARS-CoV-2 varaints- a nationwide Danish cohort study (2).docx [file pmed.1003992.s008.docx]

In this manuscript, “Vaccine effectiveness against SARS-CoV-2 infection and COVID-19-related hospitalization with the Alpha, Delta, and Omicron SARS-CoV-2 variants: a nationwide Danish cohort study”, the authors estimate vaccine effectiveness of 2 or 3 doses of mRNA vaccine (vs. unvaccinated) in three time periods (corresponding with Alpha, Delta, and Omicron pre-dominance) for two age groups for SARS-CoV-2 infection and hospitalization by days since last vaccine dose. They utilize data from multiple nation-wide registries in Denmark to identify vaccination history, SARS-CoV-2 PCR test results, hospitalizations, and demographic covariates. Overall, this work provides a very nice contribution to the VE literature by estimating VE across many dimensions (age, number of doses, variant, outcome, time since vaccination) using a large general population sample and consistent methods; however, some additional clarification is needed.

Major

----

* There is a large drop in adjusted VE for 3 doses against hospitalization for Omicron among those 18-59 from 72.8% at 91-120 days to 33.3% at >120 days that should be explained. It is also much lower than the equivalent number for 2 doses (65.9%). Why are we seeing this? Perhaps this is due to the small sample size and some selection bias (i.e., to have enough time to receive 3 doses and have > 120 days accumulate afterwards someone had to be vaccinated earlier meaning that this group may be more vulnerable overall)?

* Since ICD-10 codes were available for hospitalizations and underlying hospitalization rates may differ between the vaccinated and unvaccinated (since vaccinated tend to be older/sicker overall), would it be possible to perform a sensitivity analysis looking at VE against hospitalization using COVID-related ICD-10 codes?

Moderate

----

* A major strength of this paper is estimating so many dimensions of COVID VE using the same population and consistent methods; however, it can be hard for the reader to keep track of what is being compared. As much as possible, the authors should try to standardize the language they’re using. For example, by always using the same order for # doses, outcome, variant, age group in figure titles. Similarly, it would be nice to further combine Figures 4-7 (or make it so they are displayed on the same page) so that the reader can more easily visually compare the different estimates.

* Were any steps taken to account for timing between the doses (i.e., if someone received a third dose <4.5 months after their second dose, would they have been included)?

* Should include a methods section on the whole genome sequencing data explaining how this is collected/how many samples are sequenced, etc.

Minor

---

* A few sentences were confusing to me:

- Abstract – “Among both age groups, VE against COVID-19 related hospitalization 14-30 days since vaccination with two or three doses was 94.8% or above for the Alpha and Delta variants, whereas among the youngest age group, VE estimates against the Omicron variant after two or three doses were …”. It’s not clear why VE in both age groups for Alpha and Delta is being compared with VE for only the younger age groups for Omicron.
- Line 58-60 “A systematic review and meta-regression…”
- Line 147-149 “Vaccinated individuals were followed from the start of the study or the date of assumed protection after the second or third vaccine dose, i.e. 14 days since vaccination. All individuals remained in follow-up until the date of SARS-CoV-2 infection, booster vaccination (third dose)” – Consider clarifying that being removed after a third dose only applies to those with two doses?
- Line 172-177 “From a high proportion of all SARS-CoV-2 infections, with a median of 77% and 87% mid (<3,000 Ns) and high quality (>150 Ns) genomes were obtained during…” – Should define “Ns”, not clear how the median is taken
- Line 367-370 – Consider adding that this is “compared to two doses” or something similar

* Line 90-91 “most vulnerable citizens” – be more specific here if possible

* Line 91 “the younger population was invited later” – provide a specific date here if possible

* The authors did a nice job including relevant background information on Denmark. This section was helpful for understanding study design choices and had just the right amount of detail.

* Line 139 – it is not clear where this WGS data comes from.

* It would be helpful to add a sentence to the methods or beginning of the results section pointing out that due to the timing of vaccination programs, VE estimates aren’t available for all combinations of # of doses, age group, and variant.

* Line 165 – “In the Alpha and Delta dominant periods”

* Table 1 – is there any missing data?

* Figure 1 x-axis labels are formatted differently from the Figure 2/3 x-axis labels
