# Supplementary material for: Vaccine effectiveness against SARS-CoV-2 infection or COVID-19 hospitalization with the Alpha, Delta, or Omicron SARS-CoV-2 variant: A nationwide Danish cohort study
Source: PLoS Med. 2022 Sep 1;19(9):e1003992. doi: 10.1371/journal.pmed.1003992 (PMC9436060; doi:10.1371/journal.pmed.1003992)
Supplement: Attachment — Submitted filename: Requests.docx [file pmed.1003992.s009.docx]

Dear editors and reviewers,

Thank you for taking your time to comment on the manuscript. Your comments have been very helpful to improve the manuscript. We have added the supporting information as requested. Furthermore, we have changed the definition of COVID-19 hospitalization to include COVID-19 ICD-10 codes. The information have been added in the manuscript and a point by-point response is included below.

## Requests from the editors:

In your Abstract, please combine the Methods and Findings sections into one section, “Methods and findings”.

**Response:** The headings have been corrected as requested.

In the last sentence of the Abstract Methods and Findings section, please describe the main limitation(s) of the study's methodology.

**Response:** The main limitations of the study's methodology have been added to the methods and findings section as requested.

At this stage, we ask that you include a short, non-technical Author Summary of your research to make findings accessible to a wide audience that includes both scientists and non-scientists. The Author Summary should immediately follow the Abstract in your revised manuscript. This text is subject to editorial change and should be distinct from the scientific abstract. Please see our author guidelines for more information: <https://journals.plos.org/plosmedicine/s/revising-your-manuscript#loc-author-summary>

**Response:** a non-technical Author Summary of the research has been added to the manuscript.

Please place citations in square brackets.

**Response:** Has been changed as requested.

Many thanks for providing a STROBE checklist. Please update the checklist to use section names and paragraph numbers, rather than page/line numbers (as these are likely to change during the revision process).

**Response:** section names and paragraph numbers has been added to the checklist.

Did your study have a prospective protocol or analysis plan? Please state this (either way) early in the Methods section.

a) If a prospective analysis plan (from your funding proposal, IRB or other ethics committee submission, study protocol, or other planning document written before analyzing the data) was used in designing the study, please include the relevant prospectively written document with your revised manuscript as a Supporting Information file to be published alongside your study, and cite it in the Methods section. A legend for this file should be included at the end of your manuscript.

b) If no such document exists, please make sure that the Methods section transparently describes when analyses were planned, and when/why any data-driven changes to analyses took place.

c) In either case, changes in the analysis-- including those made in response to peer review comments-- should be identified as such in the Methods section of the paper, with rationale.

**Response:** No document exist. Methods section transparently describes the analyses.

The main text is missing a reference to Figure 3 (should the reference at line 187 read ‘3’ rather than ‘2’?).

**Response:** The reference has been corrected.

Apologies if I missed it but I can’t see the corresponding asterisk in Table 1.

**Response:** You are right. The asterisk has been added.

Figures 2/3 – Please update x-axis to read ‘oct’ rather than ‘okt’.

**Response:** The x-axis has been updated.

Tables 2-5: Please consider also including the unadjusted analyses as Supplementary Tables.

**Response:** Cox regression models is always adjusted for underlying time. However, we included the estimates from the Cox regression model without further adjustment for age, sex, comorbidity and geographical region as supplementary tables.

Please ensure journal abbreviations in the references list are consistent (e.g. 'The New England Journal of Medicine' in reference 2 ought to read ‘N Eng J Med’ as per reference 3).

**Response:** Has been corrected.

## Comments from the reviewers:

**Reviewer #1:**

This paper collates various databases to estimate vaccine effectiveness in Denmark. I was asked for a statistical report and I interpret that to include all aspects of the design and conduct of the study.

Page 6 Was the age only available in 5-year bands or did the authors decide to categorise it? Categorising an essentially continuous variable wastes information (Altman and Royston, 2006; Royston et al., 2006) and leads to models which are often implausible as they predict the effect remaining at within categories and then jumping to a new value at the category boundary.

**Response:** Thank you for raising this point. The models have now been reestimated with adjustment for continuous instead using a restricted cubic spline function with five knots with the position of the knots at the 5^th^, 27.5^th^, 50^th^, 77.5^th^ and 95^th^ percentiles of the data distribution (following the approach proposed in Regression modelling strategies by Harrell, 2001).

Page 9 I think it would be better to include more explanation in the captions to the figures so that the figure plus caption are self-explanatory and do not need cross-referencing to the main text. Readers of the article online will see thumbnails and if they click on them they are not seeing the text. In the printed version there is no guarantee that the text is near the figure either. For instance in the case of Figure 1 some explanation of the blue jagged line would be good. I know we can puzzle it out for ourselves. Most of the other figures have rather sparse captions.

**Response:** More explanation in the captions to the figures has been added.

Page 9 I think some readers aged 60 and over might object to be called elderly. `Older age groups were . . . ' might be kinder.

**Response:** We agree. The phrasing has been changed.

Page 14 Would it be helpful to present the results from these other studies in tabular form to aid comparison? If that is done then relevant comparator results from this article could be repeated there so readers can see the totality of evidence in one place.

**Response:** A table with results from previous studies has been added as supplementary. However, the previous studies demonstrate many VE estimates by time since vaccination, which is difficult to fit into one table.

Table 1 Somewhere we need an explanation for the choice of 60 years as the cut-point. Was this how the Danish health system decided to prioritise in the first wave of vaccinations?

**Response:** Thanks, that is correct. Vaccinations were rolled out according to age group. Those aged 60 and above were vaccinated earlier. We have now clarified this point under the heading *The Danish COVID-19 vaccination program* in the Methods section.

Table 1 The caption defines an asterisk (*) but I do not see it in the body of the table. Apologies if this is just my ageing eyes.

**Response:** You are right. The asterisk has been added.

*Points of detail*

As time went by the number of unvaccinated people became quite small. From the point of view of analysis this is unimportant as the confidence intervals take care of the imprecision but who were these people? If they are very different from the vaccinated people then using them as a control group becomes problematic. Is there any Danish data on what mixture they were of people with needle phobia, people with auto-immune conditions worried about the effect on their primary diagnosis, people who knew Bill Gates was going to implant a micro-chip in them, . . . ? Obviously this would have to come from outside the present study. In my country early on the true statement that we had no data on pregnant women was translated into the advice which many pregnant women received not to get vaccinated. This had tragic effects. Did this also happen in Denmark?

**Response:** Thank you. We agree with the reviewer’s point that the population that remain unvaccinated throughout the study may not be comparable to the rest of the population and may increasingly form a poor basis for comparison. We have now added this point to the limitations section in the discussion. We have now also adjusted the analyses for main comorbidities. It is hard to know, however, how vaccine scepticism might correlate with other behaviours leading to infection exposure risk. A vaccine hesitant pregnant woman, for example, might take further precautions to avoid SARS-CoV-2 than someone who believes in vaccine conspiracy theories and possibly does not even believe in COVID-19. The reviewer asks about the situation in Denmark: anecdotally we are aware of all the causes of vaccine hesitancy mentioned by the reviewer, although conspiracy theorists are likely less common than in other parts of the world.

Michael Dewey

*References*

D G Altman and P Royston. The cost of dichotomising continuous variables. British Medical Journal, 332:1080, 2006.

P Royston, D G Altman, andWSauerbrei. Dichotomizing continuous predictors in multiple regression: a bad idea. Statistics in Medicine, 25:127-141, 2006.

**Reviewer #2:**

The authors did an excellent job providing valuable vaccine effectiveness data on omicron as it compares to alpha and delta.

1) we've noticed that hospitalization is a bit of a messy definition during omicron where many cases are incidentally infected with omicron or it aggravates an underlying medical problem. UKHSA saw a lower VE against hospitalization in younger cohorts specifically where you expect more incidental covid. see this summary <https://linkinghub.elsevier.com/retrieve/pii/S0264410X22005230>

**Response:** Thank you for sharing the summary. We changed the definition of COVID-19 hospitalization so it is now based on COVID-19 ICD-10 codes.

2) can you please just include a sentence about how you dealt with persons with prior infection (were they included or excluded?) given the sample size i can't imagine it'll change the results dramatically especially for alpha/delta but just good to state.

**Response:** We did not include individuals with prior infection. This is mentioned in the methods section: Individuals with a reverse transcription polymerase chain reaction (RT-PCR)-confirmed SARS-CoV-2 infection before the start of the study periods and those without any tests during the three variant periods were excluded from the analyses

3)if there is room, for your consideration is to add in the limitations that persons are assumed to be negative in this national cohort--undiagnosed persons or persons testing positive on home-based testing (if applicable in denmark) could bias the results.

**Response:** This has been added to the discussion section.

Excellent contribution to the literature.

Minal Patel

**Reviewer #3:**

In this manuscript, “Vaccine effectiveness against SARS-CoV-2 infection and COVID-19-related hospitalization with the Alpha, Delta, and Omicron SARS-CoV-2 variants: a nationwide Danish cohort study”, the authors estimate vaccine effectiveness of 2 or 3 doses of mRNA vaccine (vs. unvaccinated) in three time periods (corresponding with Alpha, Delta, and Omicron pre-dominance) for two age groups for SARS-CoV-2 infection and hospitalization by days since last vaccine dose. They utilize data from multiple nation-wide registries in Denmark to identify vaccination history, SARS-CoV-2 PCR test results, hospitalizations, and demographic covariates. Overall, this work provides a very nice contribution to the VE literature by estimating VE across many dimensions (age, number of doses, variant, outcome, time since vaccination) using a large general population sample and consistent methods; however, some additional clarification is needed.

Major

----

* There is a large drop in adjusted VE for 3 doses against hospitalization for Omicron among those 18-59 from 72.8% at 91-120 days to 33.3% at >120 days that should be explained. It is also much lower than the equivalent number for 2 doses (65.9%). Why are we seeing this? Perhaps this is due to the small sample size and some selection bias (i.e., to have enough time to receive 3 doses and have > 120 days accumulate afterwards someone had to be vaccinated earlier meaning that this group may be more vulnerable overall)?

**Response:** The large drop in VE for three doses against hospitalization for Omicron among individuals aged 18-59 years may be explained by that this age group were not officially invited for the third dose at that time that they would have been able to contribute to >120 days since the third dose. The individuals contributing to this time period in this age group might be individuals in immunosuppressive therapy. Therefore, there is little follow-up time for this time period, which results in an uncertain estimate. The estimates were included in the manuscript by mistake in the first place, because we do not think there is enough substance for this estimate. Therefore, it only appeared in the table and not in the figure. The estimate is even more uncertain after changing the definition of covid-19 hospitalization.

* Since ICD-10 codes were available for hospitalizations and underlying hospitalization rates may differ between the vaccinated and unvaccinated (since vaccinated tend to be older/sicker overall), would it be possible to perform a sensitivity analysis looking at VE against hospitalization using COVID-related ICD-10 codes?

**Response:** Thank you for this comment. We have also become increasingly aware of the importance of a more specific definition for COVID-19 hospitalisations, especially in the Omicron period when infection became hugely prevalent in the population, including the hospital population. We therefore agree that it is better to perform the analysis for VE against COVID-19 hospitalization using COVID-19 ICD-10 codes instead of the less specific definition of COVID-19-related hospitalization. We have not included a sensitivity analysis, instead we have upgraded the analyses of hospitalisations to be further based on primary ICD-10 codes B342 or B972. This has now also been clarified in methods section.

Moderate

----

* A major strength of this paper is estimating so many dimensions of COVID VE using the same population and consistent methods; however, it can be hard for the reader to keep track of what is being compared. As much as possible, the authors should try to standardize the language they’re using. For example, by always using the same order for # doses, outcome, variant, age group in figure titles. Similarly, it would be nice to further combine Figures 4-7 (or make it so they are displayed on the same page) so that the reader can more easily visually compare the different estimates.

**Response:** We updated the table and figure titles to make it easier to follow that is being compared. Furthermore, we combined figure 4 and 5 and combined figure 6 and 5. We think the figures would be too small if we combined them all into one figure.

* Were any steps taken to account for timing between the doses (i.e., if someone received a third dose <4.5 months after their second dose, would they have been included)?

**Response:** Thanks. We have now clarified in the methods section that the analysis did not include individuals with fewer than 19 days between dose 1 and 2 or fewer than 140 days between dose 2 and 3.

* Should include a methods section on the whole genome sequencing data explaining how this is collected/how many samples are sequenced, etc.

**Response:** The information has been added to the methods section.

Minor

---

* A few sentences were confusing to me:

- Abstract – “Among both age groups, VE against COVID-19 related hospitalization 14-30 days since vaccination with two or three doses was 94.8% or above for the Alpha and Delta variants, whereas among the youngest age group, VE estimates against the Omicron variant after two or three doses were …”. It’s not clear why VE in both age groups for Alpha and Delta is being compared with VE for only the younger age groups for Omicron.

**Respone:** The results has been added for the individuals aged 60 years or above (only available for three doses).

- Line 58-60 “A systematic review and meta-regression…”

**Response:** More details has been added.

- Line 147-149 “Vaccinated individuals were followed from the start of the study or the date of assumed protection after the second or third vaccine dose, i.e. 14 days since vaccination. All individuals remained in follow-up until the date of SARS-CoV-2 infection, booster vaccination (third dose)” – Consider clarifying that being removed after a third dose only applies to those with two doses?

**Response:** Thanks. This has now been clarified.

- Line 172-177 “From a high proportion of all SARS-CoV-2 infections, with a median of 77% and 87% mid (<3,000 Ns) and high quality (>150 Ns) genomes were obtained during…” – Should define “Ns”, not clear how the median is taken

**Response:** More details has been added.

- Line 367-370 – Consider adding that this is “compared to two doses” or something similar

**Response:** Compared to two doses has been added as suggested.

* Line 90-91 “most vulnerable citizens” – be more specific here if possible

**Response:** More details has been added.

* Line 91 “the younger population was invited later” – provide a specific date here if possible

**Response:** More details has been added.

* The authors did a nice job including relevant background information on Denmark. This section was helpful for understanding study design choices and had just the right amount of detail.

* Line 139 – it is not clear where this WGS data comes from.

**Response:** The information has been added to the methods section.

* It would be helpful to add a sentence to the methods or beginning of the results section pointing out that due to the timing of vaccination programs, VE estimates aren’t available for all combinations of # of doses, age group, and variant.

**Response:** The information has been added to the methods section.

* Line 165 – “In the Alpha and Delta dominant periods”

**Response:** The sentence has been corrected.

* Table 1 – is there any missing data?

**Response:** No missing data. This information is added to the methods section.

* Figure 1 x-axis labels are formatted differently from the Figure 2/3 x-axis labels

**Response:** The labels has been corrected.

**Reviewer #4:**

This study is of obvious relevance to public health, and the methods are clearly described. It is reassuring that the efficacy of two doses against hospitalised COVID-19 in those over 60 remains moderately high even with the Omicron variant. My comments focus mainly on the methods.

1. For COVID-19 related hospitalisation as outcome, the case definition is based on first positive test from 14 days before to 2 days after admission. This excludes "probable health-care associated infections", defined by ECDC as cases having first positive test after more than 7 days in hospital. In the UK at the height of the epidemic "probable HAI" accounted for up to one-third of hospitalised COVID-19 cases and an even higher proportion of severe cases. If the proportion of "probable HCAI" was high in Denmark also, it would be important to report an analysis that includes this category in the case definition, even if only as supplementary material.

**Response:** Thank you for this suggestion, however we prefer not to include healthcare associated infections in the analyses. We believe this would complicate interpretation of the estimated vaccine protection against serious disease, which we attempt to capture through hospitalisations *for* COVID-19. To this end we have now further refined the definition of a COVID-19 hospitalisation to also require ICD-10 primary diagnosis codes B342 or B972.

2. In the Omicron wave, a high proportion of COVID-related hospitalisations may have been people hospitalised for other reasons,in whom a positive test was an incidental finding on admission. This may account for the apparently modest efficacy of 2 doses against Omicron-related hospitalisations in those aged under 60 years. The authors should discuss this possibility, and if possible try restricting the case definition to those whose hospitalisation was for COVID rather than with COVID if any further information such as main diagnosis or admitting specialty can be extracted from the electronic health record.

**Response:** We thank the reviewer for this suggestion which was also made by reviewer #3. We have now updated the analysis by only including hospitalisations with COVID-19 primary diagnoses (ICD-10 codes B342 or B972).

3. For infection as outcome, the case definition is based on detection by on-demand testing. This may introduce selection bias: for instance the detection rate may vary with vaccination status for instance where regular testing and vaccination are mandatory in occupations such as health care. This is noted in the Discussion, though the statement that "we cannot exclude time-varying factors such as test frequency" is rather confusing as the problem is not that test frequency is time-varying. It would be useful to report testing rates by vaccination status over time.

**Response:** We agree that the issue is not to do with time-varying factors - the point made in the discussion has now been clarified. Additionally, table 1 now reports testing rates by vaccine status during the three periods – thank you for this suggestion.

Most other studies of vaccine efficacy against infection have used test-negative control designs, though this may introduce other sources of bias. If the authors are able to link to negative test results as well as positive ones, it would be useful to report a test-negative control analysis for comparison with the cohort analysis. As the cohort design and the test-negative design have different biases, this would help to establish how robust are the findings with respect to infection as outcome. This applies only to the analyses with infection as outcome: for hospitalisation as outcome the case ascertainment is complete and detection bias does not arise.

**Response:** Thank you for these thoughts and suggestions. We are aware that many studies of VE against infection utilise a test-negative control design instead of a cohort design. However, we are fortunate to be able to conduct comprehensive cohort analyses through our high-quality national registries that contain complete information on positive as well as all negative tests done in the country. Although the Danish population was one of the most frequently tested populations in the world, we are aware that a small number of people refrained from being tested and that this had the potential to cause bias in the results. For this reason our analyses were restricted to only include people who were tested at least once during the three variant periods when Alpha, Delta and Omicron predominated. In effect, we don’t think a test-negative design would contribute much in terms of assuring equal test-seeking behaviour. A test-negative design would also not deal with the concern that the remaining unvaccinated population may not be comparable with the vaccinated population. We are therefore hesitant to introduce a completely different study design and set of results as suggested, even if just in the appendix, however if this is still considered an important suggestion we can reconsider.
